# Characterization of maize *roothairless6* which encodes a D-type cellulose synthase and controls the switch from bulge formation to tip growth

**DOI:** 10.1038/srep34395

**Published:** 2016-10-06

**Authors:** Li Li, Stefan Hey, Sanzhen Liu, Qiang Liu, Colton McNinch, Heng-Cheng Hu, Tsui-Jung Wen, Caroline Marcon, Anja Paschold, Wesley Bruce, Patrick S. Schnable, Frank Hochholdinger

**Affiliations:** 1 Department of Agronomy, Iowa State University, Ames, IA 50011-3650, USA; 2 INRES, Institute of Crop Science and Resource Conservation, Crop Functional Genomics, University of Bonn, 53113 Bonn, Germany; 3 Department of Plant Genetics & Breeding, China Agricultural University, Beijing 100193, China; 4 Department of Genetics, Development, and Cell Biology, Iowa State University, Ames, IA 50011-3650, USA; 5 Pioneer Hi-Bred International, Inc. - A DuPont Company, Johnston 50131-0184, Iowa, USA; 6 Center for Plant Genomics, Iowa State University, Ames, IA 50011-3650, USA; 7 Plant Sciences Institute, Iowa State University, Ames, IA 50011-3650, USA

## Abstract

Root hairs are tubular extensions of the epidermis. Root hairs of the monogenic recessive maize mutant *roothairless 6 (rth6*) are arrested after bulge formation during the transition to tip growth and display a rough cell surface. BSR-Seq in combination with Seq-walking and subsequent analyses of four independently generated mutant alleles established that *rth6* encodes CSLD5 a plasma membrane localized 129 kD D-type cellulose synthase with eight transmembrane domains. Cellulose synthases are required for the biosynthesis of cellulose, the most abundant biopolymer of plant cell walls. Phylogenetic analyses revealed that RTH6 is part of a monocot specific clade of D-type cellulose synthases. D-type cellulose synthases are highly conserved in the plant kingdom with five gene family members in maize and homologs even among early land plants such as the moss *Physcomitrella patens* or the clubmoss *Selaginella moellendorffii*. Expression profiling demonstrated that *rth6* transcripts are highly enriched in root hairs as compared to all other root tissues. Moreover, in addition to the strong knock down of *rth6* expression in young primary roots of the mutant *rth6*, the gene is also significantly down-regulated in *rth3* and *rth5* mutants, while it is up-regulated in *rth2* mutants, suggesting that these genes interact in cell wall biosynthesis.

The epidermis of plant roots comprises of two types of cells: trichoblasts and atrichoblasts. During development trichoblasts give rise to tubular extensions which develop into root hairs. Root hair formation is a three step process that is initiated by the swelling of a bulge, followed by tip growth initiation and elongation through polarized exocytosis^1^. Root hairs of cereals significantly increase the root surface and are therefore instrumental for nutrient uptake and optimal development^2^,^3^,^4^,^5^,^6^.

Different modes of epidermis differentiation into trichoblasts and atrichoblasts have been observed in plants. Trichoblast formation in Arabidopsis is position-dependent. Only epidermis cells that are situated in a cleft between two underlying cortical cells become trichoblasts and form root hairs. In Arabidopsis, the molecular network involved in epidermis specification and thus the differentiation into trichoblasts and atrichoblasts is well understood (reviewed in: Hochholdinger and Nestler^7^). In contrast, maize epidermis cells can differentiate into trichoblasts irrespective of their position^8^,^9^. In maize, *roothairless5 (rth5*) is the only gene known to date that controls root hair density and thus the differentiation of epidermis cells into trichoblasts^10^.

In maize, four genes that control root hair elongation have been identified. The *roothairless 1 (rth1*) gene encodes a SEC3 subunit^11^ of the exocyst complex which tethers exocytotic vesicles prior to their fusion thus mediating exocytotic tip growth of root hairs^12^. The *roothairless3 (rth3*) gene is translated into a monocot-specific COBRA like cell wall protein^13^. A phylogenetic study on gene families related to cell wall formation revealed that mutant alleles of *ZmCslD5* exhibit a root hair defect^14^. Most recently, the *roothairless5 (rth5*) gene was shown to give rise to a monocot-specific NADPH oxidase, which is involved in the tip growth of root hairs^10^.

Plant cell walls provide structure and determine the shape of plant cells^15^. In general, two types of cell walls can be distinguished. All dicots and most monocots develop type I cell walls. Only species of the monophyletic clade commelinids which includes the poaceae family, which comprise cereals such as maize, rice and wheat develop type II cell walls^16^. Both, type I and type II cell walls contain mostly cellulose, synthesized by cellulose synthases of the CESA gene family^14^. Cell expansion integrates the loosening of existing cell wall components with the deposition of new components. In elongating cells, cellulose microfibrils are separated and reoriented by turgor forces and subsequently interlaced by matrix constituents. In class II cell walls in species such as maize, microfibrils are crosslinked by glucuronoarabinoxylans (GAXs) while pectins contribute to only a small fraction of the matrix polymers^17^. Moreover, networks of phenylpropanoids are deposited in type II cell walls. In contrast to other commelinids, the order of poales which includes the cereals synthesize a mixed-linkage (1 → 3), (1 → 4)-β-glucan during cell expansion that is hydrolyzed when growth stops^18^. Root hairs develop two layers of cell walls. The thin α-layer at the root hair tip can be expanded and formed by turgor pressure, while the thick β-layer at the non-growing tubular parts of the root hair provides stability and strength^19^. Hence, the synthesis of cell wall components in the α-layer of root hairs is instrumental for their elongation as illustrated by mutants defective in this process^13^,^20^,^21^,^22^.

In the present study we report the cloning and functional characterization of the *rth6* gene of maize which encodes CSLD5, a monocot and root hair-specific D-type cellulose synthase required for the transition from bulge formation to tip growth in root hair formation.

## Results

### The *roothairless6* gene controls root hair length in all root-types of maize

A mutant specifically affected in root hair elongation but otherwise normal was identified in a phenotypic screen of an F_2_-population resulting from *Mutator* transposon mutagenesis. Genetic crosses established that this mutant phenotype is controlled by a single recessive allele. Moreover, genetic crosses of homozygous mutants of this new mutant allele with the previously isolated mutants *rth1* to *rth5* resulted in complementation thus demonstrating that these mutants are not allelic. Therefore, this new mutant was designated *roothairless6 (rth6*). Subsequently, additional *rth6* alleles *rth6-2* to *rth6-6* were generated (see methods).

**Figure 1:**
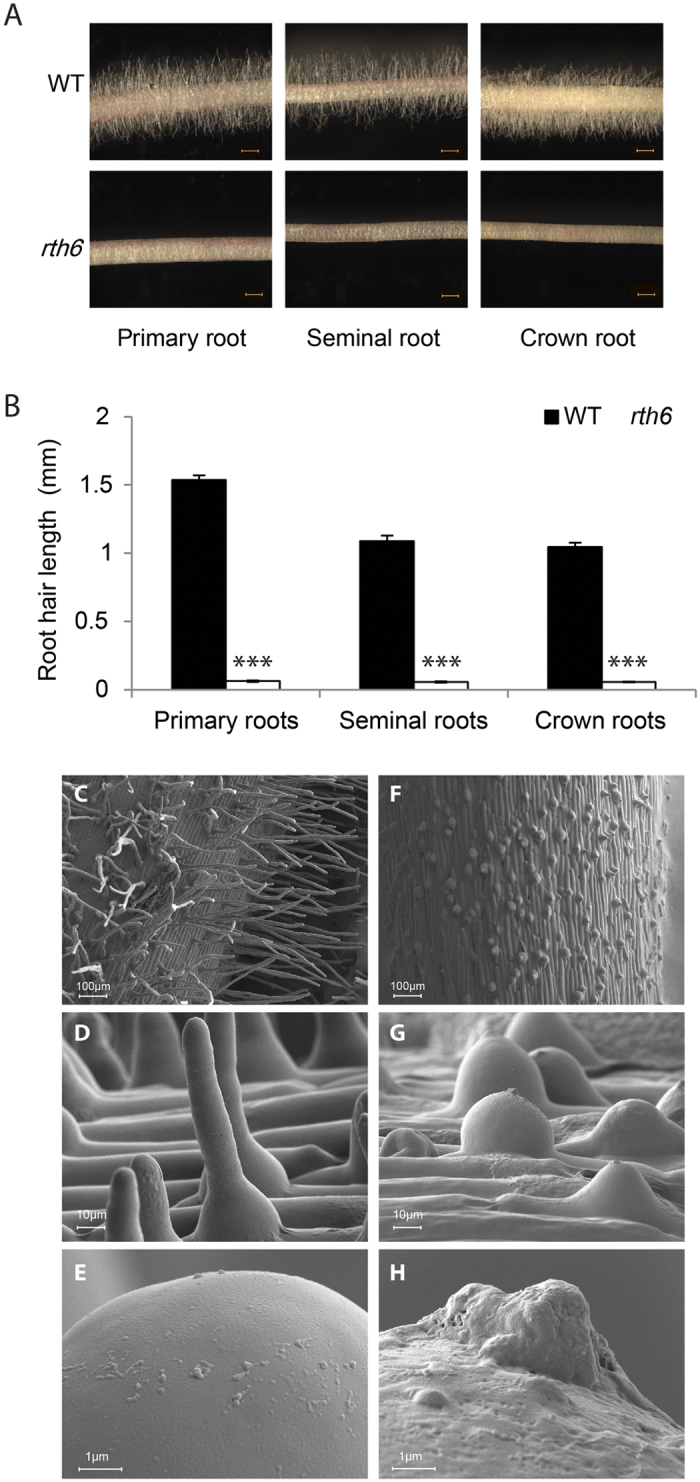
Phenotype of *rth6* mutants. (**A**) Microscopic images of 3–4 cm long primary roots, seminal roots and crown roots of wild-type (WT) (upper panels) and *rth6* mutants (lower panels). (Scale bar: 1 mm; n = 25, error bars indicate SD; Student’s t-test ***p ≤ 0.001). (**B**) Root hair length of 3–4 cm long primary roots, seminal roots and crown roots of WT and *rth6* mutants. (**C**–**H**) Cryo scanning electron microscopic (cSEM) images of WT primary root surface (**C**–**E**) and *rth6* primary root surface (**F**–**H**) at 100x magnification (**C**,**F**), 1,000x magnification (**D**,**G**) and tips of root hairs at 10,000x magnification (**E**,**H**).

Stereo microscopy demonstrated that *rth6-1* mutants display significantly shorter root hairs than their wild-type siblings in all major root-types including primary-, seminal- and crown roots (Fig. 1A). Quantification of root hair length established that on average *rth6-1* root hairs developed only 4–5% of the length of wild-type root hairs in primary, seminal and crown roots (Fig. 1B). Root hair morphology of wild-type and *rth6* was analyzed via cSEM (cryo scanning electron microscopy). High-resolution cSEM pictures revealed that *rth6* forms only root hair bulges (Fig. 1F–H) which did not elongate compared to wild-type root hairs (Fig. 1C–E). The basal bulges of *rth6* were characteristically swollen (Fig. 1G) like the basal region of wild-type root hairs (Fig. 1D). In contrast to wild-type root hairs, the bulges of *rth*6 were arrested at this developmental stage and unable to elongate. High-resolution pictures of root hair tips illustrated that wild-type root hairs had a smooth surface (Fig. 1E) while the surface of mutant *rth6* root hairs was rough as a consequence of defects during root hair formation (Fig. 1H). In addition, mutant *rth6-1* root hair tips (Fig. 1H) displayed small outgrowths with a very rough surface at the tip. These are likely the initiation sites which represent the switch from bulge formation to root hair elongation which is defective in *rth6-1*.

### Cloning of *rth6* by a combination of BSR-Seq and Seq-walking

**Figure 2:**
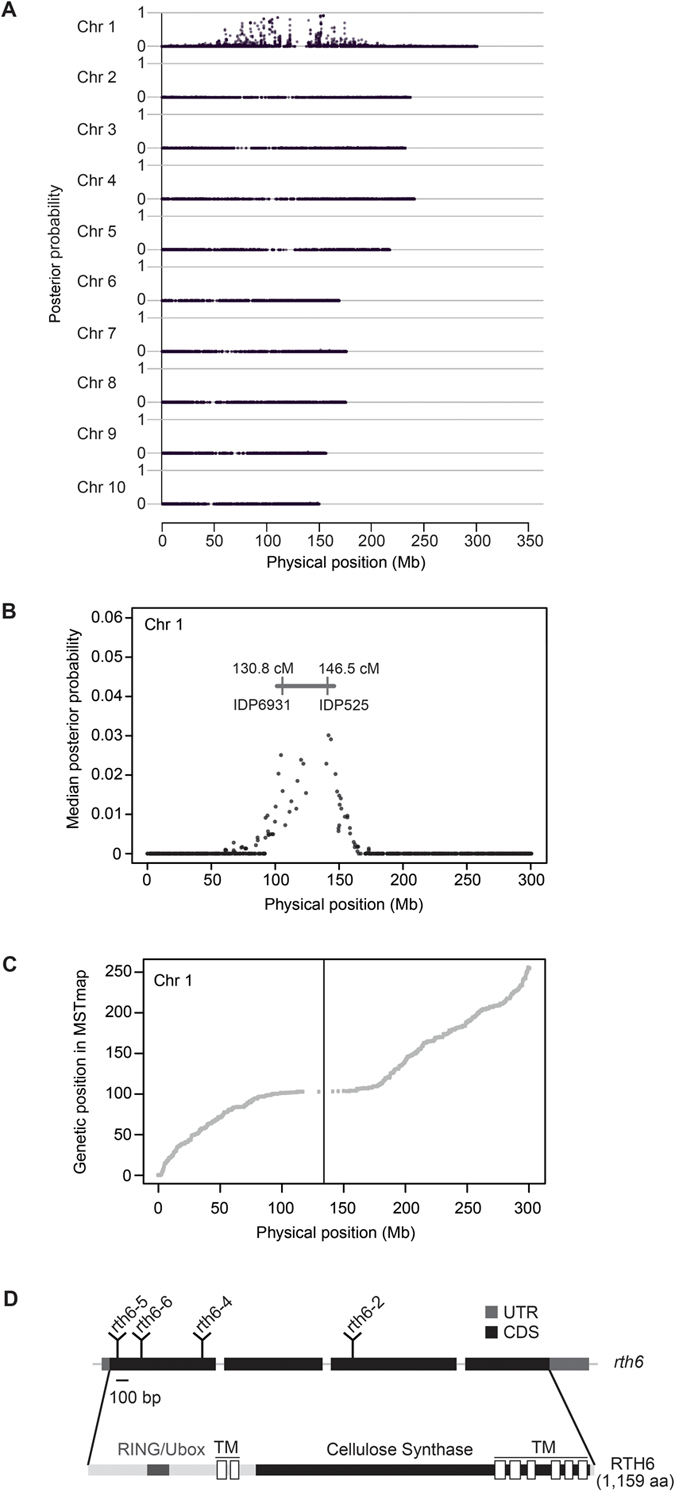
BSR-Seq mapping of *rth6*. (**A**) BSR-Seq analyses confines *rth6* to the centromeric region of chromosome 1. (**B**) BSR-Seq scanning defines a 15.7 cM *rth6* interval which corresponds to ~50 Mb. (**C**) Correlation of physical and genetic map positions illustrate the low recombination rates in the centromeric region of chromosome 1. The vertical line indicates the position of the *rth6* gene on the physical map. (**D**) Gene structure of *rth6*. Exons: black boxes, introns: thin lines between black boxes, UTR: untranslated region, CDS: coding sequence. The positions of transposon insertions in for novel alleles, *rth6-2, rth6-4, rth6-5* and *rth6-6* are indicated. Functional domains of the RTH6 protein are indicated. RING/Ubox: RING/Ubox like Zinc-finger domain, TM: transmembrane domain.

The *rth6* gene was genetically mapped to the short arm of chromosome 1 by B-A translocation stocks. Subsequently, 123 mutant and 123 wild-type individuals of a F_2_-mapping population segregating for *rth6* mutant phenotypes were subjected to BSR-Seq. This analysis demonstrated that the causative gene maps to the centromeric region of chromosome 1 (Fig. 2A). Chromosome 1 was then scanned using a window containing 100 SNPs and a step size of 20 SNPs. Within each window, the median linkage probability obtained from a Bayesian BSA analysis across all 100 SNPs was determined and plotted against the middle physical position of the window. This process mapped the *rth6* gene to a 15.7 cM interval flanked by IDP (insertion deletion polymorphism) markers IDP6931 (130.8 cM) and IDP525 (146.5 cM) of ISU IBM Map7 1 (Fig. 2B) which corresponded to ~50 Mb on the physical map of chromosome 1 (ZmB73_AGPv1 release 4a53; http://ftp.maizesequence.org/release-4a.53/). Correlation of physical and genetic distances on chromosome 1 calculated via molecular markers from an IBM genetic map^23^ indicated that the centromeric region denoted by a vertical line exhibited low rates of recombination per Mb (Fig. 2C).

Genomic DNA extracted from the inbred line B73 and a pool of seedlings derived from a self of a plant with the genotype *rth6-1*/*rth6-2* was used to construct Seq-walking libraries, which were then sequenced on an Ion Proton instrument (see methods). In total, 16,680,521 raw sequence reads were obtained, 7,175,209 from the *rth6* library and 9,505,312 from the B73 library. Reads were sorted and decoded according to their barcode sequences. Subsequently, barcodes, adapters and *Mu*-related sequences were removed. The remaining *Mu* flanking sequences that were longer than 90 bp (3,054,970 and 2,703,957, respectively) were aligned to the B73 reference genome.

*Mu* insertions recovered from the B73 Seq-Walking library were assumed not to be responsible for an *rth6* mutation. This subtractive process resulted in the identification of 29 *Mu* insertion sites within the *rth6* mapping interval (Chr1: from 102, 196, 700 bp to 144, 956, 100 bp) that had been defined by the BSR-Seq experiment (Fig. 2B). Many more reads (N = 22,945) were obtained for a single insertion site (Chr1: 104, 610, 900 bp) within the GRMZM2G436299 gene than for any other of the 28 sites within the mapping interval (Table S1). Confirmative PCR experiments on genomic DNA of the *rth6-1* and *rth6-2* alleles demonstrated that the observed *Mu* insertion derived from allele *rth6-2*, whereas the reference allele *rth6-1* did not contain a detectable *Mu* transposon insertion. Based on the *Mu* insertion in exon 3 of *rth6-2*, we declared GRMZM2G436299 an *rth6* candidate gene.

### Confirmation of *rth6* identity via the generation of independent alleles

To confirm that GRMZM2G436299 indeed represents the *rth6* gene, two independent *Mu*-induced mutant alleles (*rth6-3* and *rth6-4*) and two *Ac*/*Ds* insertion alleles (*rth6-5* and *rth6-6*) obtained via a reverse genetic screen (see methods) were analyzed. All four of these novel alleles displayed the roothairless phenotype and transposon insertion sites were mapped by PCR and subsequent sequencing (Table 1). These experiments confirmed that GRMZM2G436299 is the *rth6* gene by independent mutant alleles which contain transposon insertions at different positions of exon 1 (*rth6-4, rth6-5, rth6-6*) and exon 3 (*rth6-2*) of the candidate gene. These results were further substantiated by crosses of the transposon induced alleles to the reference allele *rth6-1*, each of which failed to complement.

### Structure and functional domains of RTH6

The *rth6* gene contains four exons and three introns encoding a 3,866 bp open reading frame which translates into a 1,159 aa protein (Fig. 2D) with a predicted molecular weight of 129 kD and an isoelectric point of 7.9 (http://web.expasy.org). RTH6 is predicted to be a membrane protein with 8 transmembrane domains that contains an N-terminal RING/Ubox like Zinc-finger domain, and a C-terminal cellulose synthase-like protein domain (http://www.ebi.ac.uk/Tools/hmmer).

### Phylogenetic reconstructions reveal the presence of RTH6 homologs in early land plants and strict separation of mono and dicot clades

**Figure 3:**
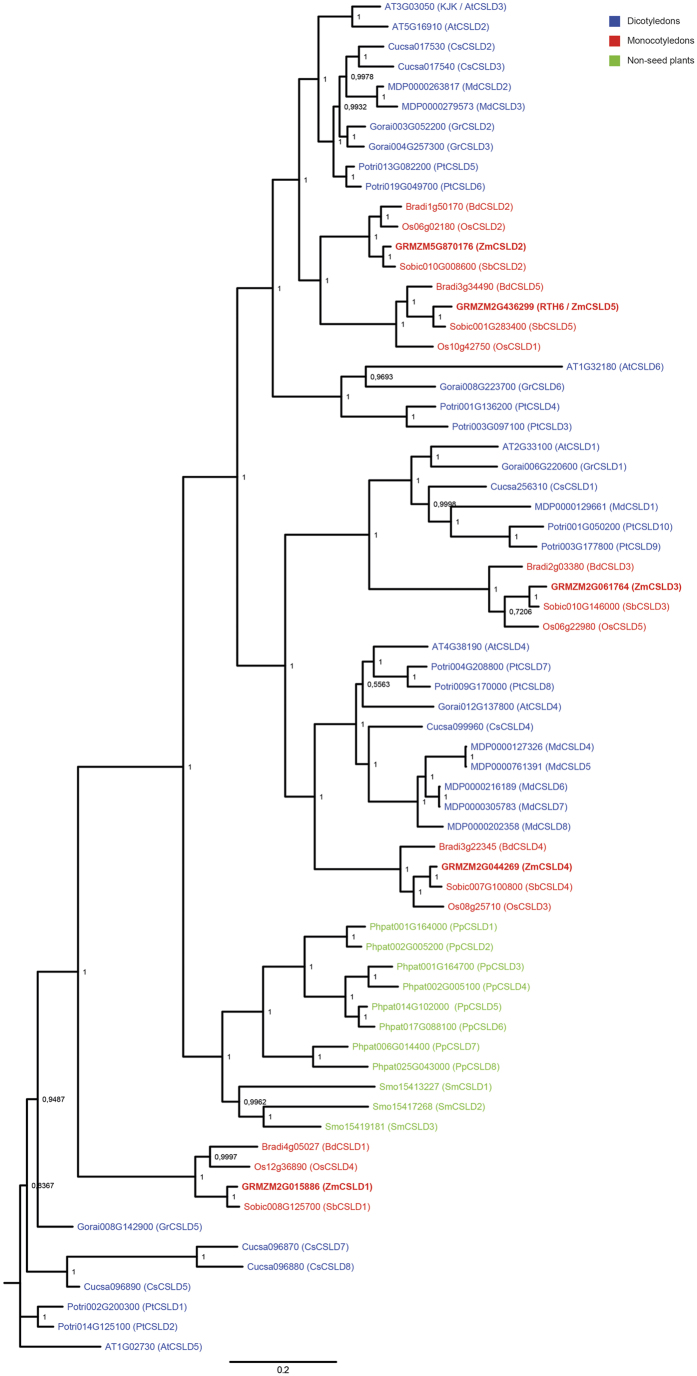
Phylogenetic reconstruction of the CSLD subfamily of Cellulose Synthases. Dicot species: blue, monocot species: red, non-seed plants: green. Posterior probabilities are indicated at the branching points.

Sequence similarity searches and phylogenetic analyses revealed that *rth6* encodes a CELLULOSE SYNTHASE-LIKE D (CSLD) protein, which belongs to the D-type subfamily of the cellulose synthase superfamily. The CSLD proteins which are illustrated in Fig. 3 cluster into monocot (Fig. 3, red), dicot (Fig. 3, blue) and non-seed-plant (Fig. 3, green) subclades which are strictly separated. Each of the analyzed monocot species maize, rice, sorghum and brachypodium encodes five *CslD* genes. The five maize *CslD* genes have been previously designated *CslD1* to *CslD5*^14^. The *rth6* gene corresponds to *CslD5*. Proteins encoded by monocot *CslD* genes form five groups in which each of the four species is represented by one protein. Typically, clusters of monocot CSLD proteins group with clusters of dicot CSLD proteins. Even evolutionary distantly related plant species such as the moss *Physcomitrella patens* or the clubmoss *Selaginella moellendorffii* encode for CSLD proteins with a unexpectedly high degree of sequence identity of 67–68% with RTH6 of maize. No homologs were identified in green algae such as *Chlamydomonas reinhardii*.

### Root-type and tissue specific expression of *rth6*

**Figure 4:**
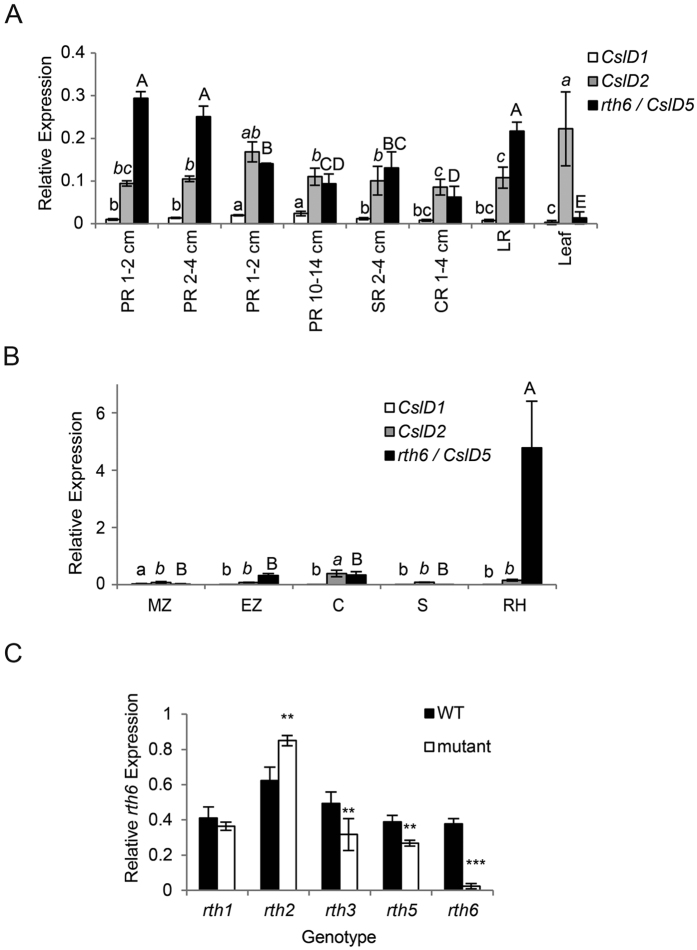
Expression of *rth6.* Expression of *CslD1* (white bars), *CslD2* (grey bars) and *CslD5*/*rth6* (black bars) in (**A**) primary roots (PR) at different developmental stages, seminal roots (SR), crown roots (CR), lateral roots (LR) and leaves, (**B**) in the meristematic zone (MZ), elongation zone (EZ), cortex (**C**), stele (S) and root hairs (RH) of 2–4 cm primary roots. (**C**) Expression of *rth6* in 2–4 cm primary roots of the mutants *rth1, rth2, rth3, rth5* and *rth6* and their wild-type (WT) siblings. Bars indicate mean relative expression ±SD (n = 4, ten plants per biological replicate). Different letters in (**A**) and (**B**) indicate significance levels at *p* ≤ 0.05 after ANOVA. Small normal letters: *CslD1*; small italics letters: *CslD2*; capital letters: *rth6*/*CslD5*. No differential expression was found for *CslD2* in (**A**). (**C**) Student’s t-test (**p ≤ 0.01; ***p ≤ 0.001).

Expression of *rth6* was surveyed in a wide range of tissues and root types by qRT-PCR (Fig. 4). In primary roots of different lengths *rth6* displayed the highest expression in young primary roots of 1–2 cm and 2–4 cm, while significantly less expression was detected in older primary roots of 4–8 cm and 10–14 cm length (Fig. 4A). Lateral roots displayed similarly high expression levels as young primary roots, whereas seminal and crown roots showed lower expression levels similar to older primary roots (Fig. 4A). Expression of *rth6* in leaves as an example for non-root tissues, was significantly lower than in most root tissues (Fig. 4A).

Primary roots of 2–4 cm length were then dissected longitudinally into the meristematic zone (MZ), the elongation zone (EZ) and the differentiation zone. The differentiation zone was mechanically dissected into the cortical parenchyma (C) comprising all cell types between epidermis and endodermis and the stele (S). Finally, root hairs (RH) of the differentiation zone were collected separately. Root hairs displayed significantly higher *rth6* expression than all other tissues. Expression in elongation zone and cortical parenchyma could be attributed to trichoblasts present in these tissues (Fig. 4B).

Finally, *rth6* expression was monitored in primary roots of 2–4 cm length in wild-type and mutant *rth1, rth2, rth3, rth5*, and *rth6* seedlings (Fig. 4C). Expression of *rth6* in mutant primary roots was on average reduced to 6% of the expression levels in wild-type primary roots. Moreover, *rth6* expression was reduced in roots of the mutants *rth3* and *rth5* and significantly up-regulated in the mutant *rth2*. Expression of *rth6* was unaffected in the mutant *rth1*. Among the five closely related *CslD* genes of maize only *CslD1, CslD2* and *CslD5 (rth6*) were expressed in roots (Fig. 4A). While, for instance *CslD2* displayed similar expression in older primary roots, seminal roots and crown roots as did *rth6* (Fig. 4A), *rth6* was the only *CslD* gene that displayed highly specific expression in root hairs (Fig. 4B).

## Discussion

Root hair length in maize *rth6* mutants is reduced to 4–5% of wild-type seedlings in all root types including embryonic primary and seminal and postembryonic shoot-borne roots. Similarly, the maize mutants *rth1*^11^, *rth2*^24^, *rth3*^13^ and *rth5*^10^ also constitutively display defects in root hair formation in all root types. In contrast, other aspects of maize root development such as lateral root formation are controlled by root-type specific genetic programs. This is illustrated by the mutants *rum1*^25^,^26^ and *lrt1*^27^. In both mutants only embryonic primary and seminal roots are defective in lateral root initiation while this process is not impaired in postembryonic shoot-borne roots.

The maize roothairless mutants identified thus far are affected at different stages of the three-step process of root hair development^1^. Both, the *rth3* mutant^13^ and the *rth6* mutant characterized in the present study can form a bulge but are unable to initiate root hair elongation. In contrast, the *rth5* mutant is impaired in cell wall loosening at the root hair tip for subsequent turgor driven tip growth^10^. Therefore, *rth5* forms a bulge and initiates tip growth but ceases elongation soon after. Finally, the mutants *rth1*^11^ and *rth2*^24^ display the longest root hairs of all identified maize roothairless mutants. In both mutants root hairs initiate polar tip growth and elongate to a certain extent, but their overall final length lags significantly behind their wild-type siblings. This diversity of root hair mutant phenotypes illustrates the subtle multi step genetic regulation of this developmental process in maize.

Molecular cloning revealed that *rth6* encodes CSLD5 of the Cellulose synthase like D (CSLD) protein family^14^. Mutants of several members of this gene family including *maize csld5*^14^, rice *csld1*^22^ and *Arabidopsis csld3/kjk*^20^,^21^, and *csld2*^28^ have been demonstrated to be defective in root hair elongation. RTH6 is predicted to contain a C-terminal cellulose synthase domain and eight transmembrane domains. Based on shared sequence identity, all members of the cellulose synthase superfamily are predicted to be membrane bound processive glycosyltransferases that synthesize b-linked glycan polymers such as cellulose or hemicellulose backbones found in cell walls^29^. Glucan chains are synthesized at the plasma membrane by cellulose syntheses complexes to be extruded and deposited at the inside of the cell wall^15^,^30^. Evidence that CSLD proteins indeed act as cellulose synthases was provided by the complementation of the Arabidopsis *csld3* mutant by a chimeric CSLD3 protein containing a CESA domain known to catalyze cellulose synthesis^31^. The N-terminal RING/Ubox Zinc-finger-like domain predicted in RTH6 is specific for the CSLD and CESA subfamilies of Cellulose Synthases^29^. This domain was suggested to mediate protein-protein interactions^32^,^33^.

Several members of the CSLD gene family have been functionally characterized and it has been demonstrated that these genes are involved in the tip growing processes. For example, *AtCslD1* and *AtCslD4*^28^, have been shown to be involved in pollen tube growth. Moreover, *AtCslD2* and *AtCslD3/kjk* have divergent and redundant functions in in root hair development and female gametophyte development^34^. Finally, ZmCSLD1 is involved in the establishment of new cross walls during cell division^35^.

Despite the high degree of sequence conservation on the amino acid level, mutations in different members of the CSLD family result in morphologically different root hair phenotypes. For instance, the maize mutant *rth6 (csld5*) and Arabidopsis *csld3*/*kjk* display very short root hairs which are arrested shortly after initiation and are unable to elongate after bulge formation. In contrast, root hairs in the rice mutant *csld1* are initiated normally but their elongation is impaired relatively late in development and root hairs display kinks and swellings along their length^22^. Hence, despite the fundamental structural differences in type I (Arabidopsis) and type II (maize and rice) cell wall compositions^16^ these closely related CSLD proteins have similar functions in root hair formation.

CSLD proteins belong to one of ten subfamilies of the highly conserved cellulose synthase superfamily^14^. The high degree of evolutionary conservation of this subfamily is supported by the observation that early land plants such as the clubmoss Selaginella and the moss Physcomitrella contain *CslD* genes (Fig. 3). Selaginella belongs to the oldest living vascular plant division Lycopodiophyta which emerged ~410 million years ago. Phylogenetic reconstruction revealed that monocot, dicot, and non-seed-plant subclades are strictly separated which might illustrate that diversification of the gene family mainly occurred along the boundary of monocot, dicot, and non-seed plant species (Fig. 3). Therefore, a one-to-one correlation of monocot and dicot CSLD proteins is difficult although it has been demonstrated that monocot and dicot members of closely related clades can have similar functions as illustrated above for the monocot genes *rth6 (ZmCsld5*) and *OsCSLD1*^22^ and the dicot genes *AtCSLD2*^28^ and *AtCSLD3/KJK*^20^,^21^ in root hair development.

Root hair specific expression of *rth6* and the visible mutant phenotype implies that *rth6* has only limited functional redundancy with other members of the maize *CslD* family. Similar root hair specific expression and visible root hair defective mutant phenotypes have been observed for the rice *OsCslD1*^22^ and maize *rth5*, which encodes a NADPH oxidase^10^, also implying limited functional redundancy with other members of these gene families. For the Arabidopsis AtCSLD2 and AtCSLD3 proteins, partial redundancy has been observed because double mutants of these genes display even shorter root hairs than the single mutants^28^.

Based on their low (*CslD2*) to absent (*CslD1, CslD3, CslD4*) expression in root hairs, the other four members of the maize *Cellulose synthase-like D* subfamily likely have other functions than controlling root hair formation. To date other than *rth6* (Zm*CslD5*), only Zm*CslD1* has been characterized by a mutant which is defective in cell division and expansion and therefore displays a significantly reduced width of several organs including leaves^14^,^35^.

**Figure 5:**
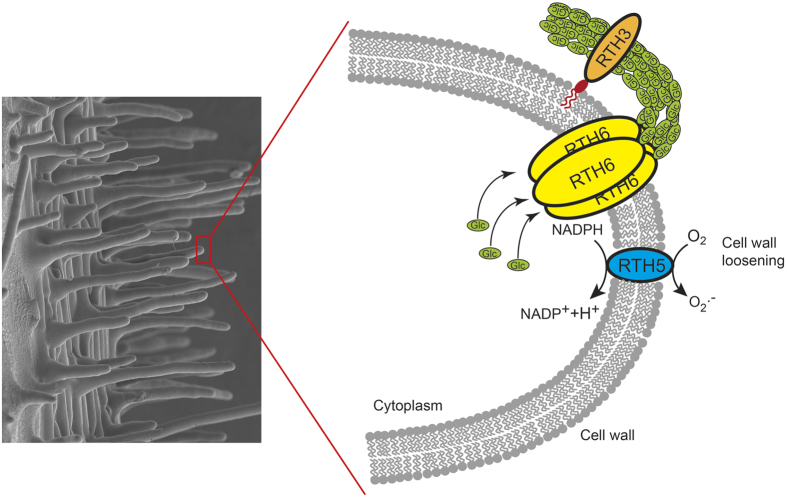
Model of the functional link of RTH3, RTH5 and RTH6 in root hair formation in maize. RTH5 produces apoplastic superoxide, which results in cell-wall loosening via hydroxyl radicals and also controls the expression of cellulose biosynthesis genes^10^. After cell wall loosening, the transmembrane protein RTH6 is arranged in rosettes and synthesizes cellulose at the plasma membrane^47^ which is extruded to the inner side of the cell wall in the root hair tip and thus reinforces the tubular shaft, while the GPI-anchored COBRA-like cell wall protein RTH3^13^ is involved in the organization of the synthesized cellulose. Glc: Glucose.

A comparative expression survey of *rth6* transcripts in wild-type versus known maize roothairless mutants revealed that the *rth2, rth3* and *rth5* genes are differentially expressed in wild-type versus mutant *rth6* primary roots. While the *rth2* gene has not yet been cloned, functional links between *rth3, rth5* and *rth6* can be established. In a comparative RNA-Seq analysis of wild-type versus mutant *rth5* roots only two GO terms were enriched^10^. In addition to “oxidation/reduction” which included some peroxidases which likely act downstream of RTH5 and facilitate the loosening of the elongating root hairs, the GO subgroup “cellulose biosynthesis” was overrepresented. Cellulose biosynthesis follows peroxidase mediated cell wall loosening^10^. Similarly, *rth3*^13^ and *rth6* are functionally linked by secondary cell wall formation. Secondary cell wall formation is a two-step process of synthesis and organization^36^,^37^. The *rth3* gene encodes a COBRA-like cell wall protein^13^. COBRA (COB) has been identified as a putative regulator of cellulose synthesis^38^. Recently, it has been suggested in Arabidopsis that COBRA and the cellulose synthase complex reside in close proximity on the plasma membrane and that COBRA facilitates cellulose crystallization from the emerging β1-4-glucan chains by acting as a “polysaccharide chaperone”^39^. Consistent with these suggested functions, RTH6 synthesizes cellulose at the plasma membrane of the root hair tips and thus reinforces the tubular shaft, while RTH3 is involved in the organization of the synthesized cellulose. Hence, defects in either of both processes lead to non-functional cell walls which explains the highly similar phenotypes of *rth3* and *rth6* mutants. The functional relationship of RTH3, RTH5 and RTH6 has been summarized in the model in Fig. 5.

## Methods

### Isolation of the reference allele *rth6-1*

The mutant *rth6* was initially identified by screening segregating F_2_-families derived from *Mu* active lines of the collection at Pioneer-Hybrid. The reference allele was designated *rth6-1* (Schnable Lab Ac #: 1351) and backcrossed into the inbred line B73 >8 times.

### cSEM imaging

Wild-type and *rth6* mutant seeds for cSEM (cryo scanning electron microscopy) were germinated for 3–4 days. Seedling roots were cut into 1–2 cm pieces with a razor blade and mounted on a specimen holder with a mixture of Tissue-Tek^®^ O.C.T.™Compound (Sakura Finetek Europe B.V., Alphen aan den Rijn, Netherlands) and colloidal graphite (Agar Scientific, Stansted, UK) and were immediately frozen in a nitrogen slush. Specimen were then transferred into a Quorum PP3010T cryo preparation chamber (Quorum Technologies, Laughton, UK) at −140 °C the specimen holder was heated to −80 °C for 40 min were water was sublimated. Subsequently, specimen were platinum sputtered at 10 mA for 60 sec and imaged in a Zeiss SIGMA VP cryo scanning electron microscope (Zeiss, Oberkochen, Germany). Images were taken at a magnification of 100x, 1,600x and 16,000x.

### BSR-Seq mapping

A line carrying the *rth6-1* allele backcrossed for five generations into the inbred line B73 was crossed with the inbred line Mo17 and subsequently self-pollinated to generate multiple F_2_-families. Kernels from one of the resulting F_2_-families that were segregating for the *rth6-1* allele were rolled up in water-soaked germination paper (Anchor paper, Roseville, MN, USA) and placed in a 10 L bucket filled with ca. 2 L of distilled water and incubated at 25 °C in dark. Subsequently, 3 cm primary roots were harvested from both the *rth6* mutants and their wild-type siblings. In total, 123 mutant and 123 wild-type individuals were collected and separately pooled according to their phenotypes. RNA was extracted separately from each pool with the RNeasy mini kit (Qiagen, Hilden, Germany) and subsequently subjected to a DNaseI treatment as per the manufacturer’s protocol. RNA quality (RIN > 8) was checked on a Bioanalyzer 2100 (Agilent, Santa Clara, CA, USA) using a RNA 6000 Nano chip. RNA-Seq libraries were constructed using the Illumina Truseq RNA-Seq sample preparation kit according to the manufacturer’s protocol. Two different Illumina TruSeq barcodes were used for the two libraries^24^. The libraries were pooled and sequenced on an Illumina HiSeq2000 (Illumina, San Diego, CA, USA) sequencer, generating 99 bp single-end reads, which were analyzed as previously described^24^. Briefly, the two alleles of a given SNP site should be detected in approximately equal numbers of RNA-seq reads when considering both pools of RNA-seq reads. In contrast, only one allele of a SNP that is completely linked to the causal gene should be present in the RNA-seq reads from the mutant pool. Hence, linkage probabilities of each SNP with the causal gene were calculated and plotted versus the physical position of the gene. Subsequently, chromosome 1 was scanned by using a chromosome window containing 100 SNPs and the median linkage probability was plotted versus the middle physical position of the window. The window was slid with a step size of 20 SNPs. Sequence reads from the BSR-Seq experiment have been deposited in the SRA (sequence read archive) under AC: SRP044758.

### Seq-Walking

A Seq-Walking library was generated from genomic DNA extracted from 24 mutant seedlings obtained via the self-pollination of a mutant plant with the genotype *rth6-1/rth6-2*. The isolated DNA was sheared by a BioRuptor-UCD-200 (Diagenode, Denville, NJ, USA) sonication system with 15 s/30 s on/off cycles at low speed. The library was prepared as described previously^24^ and sequenced on an Ion Proton system (Life Technologies, Grand Island, NY, USA). As a control, Seq-Walking was also conducted on DNA isolated from the inbred line B73. The Seq-Walking library was prepared using the itp-Mu8 (GTCGAT) barcode, while the itp-Mu31 (CTGCTA) barcode was used for B73 library generation. Other primers used for Seq-Walking library preparation have been published previously^23^.

### Confirmation of the *rth6* candidate gene by independent transposon insertion alleles

Novel *Mutator* insertions in the *rth6* gene were generated by a direct transposon-tagging experiment in which plants homozygous for the *rth6-1* allele were crossed as males with *Mu*-active stocks. Three novel alleles *rth6-2 (rth6-Mu 10B-668*), *rth6-3 (rth6-Mu 11B-451*) and *rth6-4 (rth6-Mu 11B-453*) were identified in a forward genetic screen for roothairless phenotypes among ~94,000 seeds of the resulting progeny. Mutant progeny were self-pollinated and in parallel crossed with plants homozygous for *rth6-1* to validate the new mutant alleles. Moreover, two *Ac*/*Ds* insertion alleles *rth6-5* (Plant GDB Ac#: *Ac*.mon00102; Schnable Lab Ac#: 5972) and *rth6-6* (Plant GDB Ac#: *Ds* I.S07.1244A; Schnable Lab Ac#: 5971) were obtained from Plant GDB (http://www.plantgdb.org/prj/AcDsTagging/v2/genes.php) by reverse genetic analysis. All *Mutator* and *Ac/Ds* insertion sites were mapped by PCR and subsequent sequencing using transposon-specific oligonucleotide primers in combination with the *rth6* specific oligonucleotide primers listed in Table S2.

### qRT-PCR

For quantitative real time PCR, seedlings of the maize inbred line B73 plants were grown for three to ten days in germination paper rolls (Anchor Paper) as previously described^40^ under a 16 h light/8 h dark photoperiod at 28 °C and 24 °C, respectively. Subsequently, roots of ten plants were collected for each of the four biological replicates per tissue or developmental stage and immediately frozen in liquid nitrogen. Furthermore, four different tissues of young primary roots were collected. The meristematic zone comprising the first two mm of the root tip and the proximal elongation zone were cut with a razor blade under a stereo microscope (Zeiss Stemi 2000, Zeiss). The differentiation zone which was distinguished from the neighboring elongation zone by the presence of root hairs was separated into cortical parenchyma and stele tissues as previously described^41^. For each root hair sample, 100–150 primary roots of three-day-old seedlings were dipped into liquid nitrogen. Subsequently, frozen root hairs were broken off the primary root using a pre-cooled spatula and collected in a mortar where they were pulverized in liquid nitrogen using a pestle. Total RNA was extracted with the RNeasy kit (Qiagen). RNA integrity was measured using an Agilent 2100 Bioanalyzer and a RNA 6000 Nano chip (Agilent Technologies Inc., Santa Clara, USA). RIN values for all samples were >9.5. cDNA was synthesized from 1 μg of total RNA using the Quanta qScript™ cDNA SuperMix (Quanta, Gaithersburg, MD, USA). The cDNA was 1:2 diluted with water and a dilution series was prepared up to 1:128. Each biological replicate was measured in a BioRad CFX 384 Real-Time System (Biorad, Hercules, CA, USA) in three technical replicates using the Quanta PerfeCTa^®^ SYBR^®^ Green SuperMix (Quanta). Primer efficiencies were calculated using the following formula: PCR amplification efficiency = 10^−1/slope^ − 1^42^. Primer efficiencies were between 85% and 105% and R^2^ was >0.995. Expression levels were calculated relative to a homolog of a *myosin* heavy-chain gene (GenBank accession AI941656) previously used as a reference for expression in maize roots^43^.

### Phylogenetic analysis

The predicted amino acid sequence of RTH6 was compared via tblastn against the translated nucleotide databases of *C. reinhardii, P. patens, S. moellendorffii, A. thaliana, G. raimondii, M. domestica, P. trichocarpa, C. sativus, S. bicolor, B. distachyon, Z. mays* and *O. sativa* from the Phytozome 10.1 plant genomics portal (http://phytozome.jgi.doe.gov/pz/portal.html). Homologous sequences were downloaded and aligned using ClustalW with a gap opening penalty of 10 and gap extension penalty of 0.1 using MEGA 6^44^. Alignments were exported into a NEXUS file and trees were generated by MrBayes^45^,^46^. The tree was calculated using a Markov Chain Monte Carlo approach and three hot chains and 5 million generations until the standard deviation of split frequencies dropped below 0.01. The phylogenetic tree was built using Fig Tree software (http://tree.bio.ed.ac.uk/software/figtree/).

## Additional Information

**How to cite this article**: Li, L. *et al*. Characterization of maize *roothairless6* which encodes a D-type cellulose synthase and controls the switch from bulge formation to tip growth. *Sci. Rep.* **6**, 34395; doi: 10.1038/srep34395 (2016).

## Supplementary Material

Supplementary Information

## Figures and Tables

**Table 1:** Mutant alleles of *rth6.*

Allele name	Allele ID	Type of mutation	Mutation site in bp on chr. 1 (AGPv2)
*rth6-1*	*rth6-ref*	n. d.^a^	n. d.
*rth6-2*	*rth6-Mu 10B-668*	*Mu1*	104,610,915
*rth6-3*	*rth6-Mu 11B-451*	n. d.	n. d.
*rth6-4*	*rth6-Mu 11B-453*	*Mu1*	104,609,772
*rth6-5*	*rth6-Ac*.mon00102	*Ac*	104,609,104
*rth6-6*	*rth6-Ds* I.S07.1244A	*Ds*	104,609,449

^a^n.d.: not determined.
