# Supplementary material for: Characterization of maize roothairless6 which encodes a D-type cellulose synthase and controls the switch from bulge formation to tip growth
Source: Sci Rep. 2016 Oct 6;6:34395. doi: 10.1038/srep34395 (PMC5052636; doi:10.1038/srep34395)
Supplement: Supplementary Information [file srep34395-s1.pdf]

Characterization of maize *roothairless6* which encodes a D-type cellulose synthase and controls the switch from bulge formation to tip growth

Li Li, Stefan Hey, Sanzhen Liu, Qiang Liu Colton McNinch, Heng-Cheng Hu, Tsui-Jung Wen, Caroline Marcon, Anja Paschold, Wesley Bruce, Patrick S. Schnable, Frank Hochholdinger

Supplementary Tables 1 and 2

Table S1. Number of *rth6*-specific Seq-Walking reads recovered per *Mu* insertion site in the mapped interval on chromosome 1 based on maize reference genome AGPv2.

***Mu* sites (bp)**	**No. of reads**
102,196,700	3
102,580,900	5
102,626,800	1
103,419,600	21
104,610,900	22,945
105,325,800	10
105,765,800	3
106,045,500	14
111,201,400	1
111,201,500	11
112,896,100	2
113,445,900	1
115,219,500	31
116,723,900	1
119,214,500	1
124,691,700	44
126,516,200	3
126,775,900	1
129,262,600	2
131,559,500	10
133,526,300	1
138,592,600	4
138,614,400	30
138,952,700	37
140,962,500	43

Table S2: Oligonucleotide primers used in this study.

**Flanking IDP Markers:**	
IDP6931-Fw	5´ TTGCATGTAGTGGGAAGTGC 3´
IDP6931-Rv	5´ TTAAATGGGCGAACATAGCC 3´
IDP525-Fw	5´ TATGGGAGTGAAAGCCATCC 3´
IDP525-Rv	5´ GCGTGCTGAGACTTACCTGG 3´
***Mutator* alleles:**	
MuTIR	5´ AGAGAAGCCAACGCCA(AT)CGCCTC(CT)ATTTCGTC 3`
rth6-2 (rth6C1R4)	5´ GCATGTTGACGTCGAAGAAA 3´
rth6-4 (rth6C1R2)	5´ ACTCCAGGGAGATCCGACTT 3´
***Ac/Ds* alleles:**	
Ac/Ds specific (JSR05)	5´ CGTCCCGCAAGTTAAATATGA 3´
rth6-5 (rth6C1R1)	5´ CTCCCACTCGGTGTTCTTGT 3´
rth6-6 (I.S07.1244A-R)	5´ CCCACTCGGTGTTCTTGTAAGG 3´
**qRT-PCR**	
ZmCslD1-Fw	5´ CGGTGGAGCTTCTTGATG 3´
ZmCslD1-Rev	5´ AGGAGGGCTGATGTAGAC 3´
ZmCslD2-Fw	5´ CAGCCGCACCATCTACAG 3´
ZmCslD2-Rev	5´ GTTGATGGCCACCCACAG 3´
ZmCslD3-Fw	5´ CCGTTGGCAGTGATCGGTA 3´
ZmCslD3-Rev	5´ CCAGCACCCAGAAGCTGAA 3´
ZmCslD4-Fw	5´ TCGTTTGGTCGGGACTCATC 3´
ZmCslD4-Rev	5´ AAGCTAGGCGGTCGTTGTC 3´
ZmCslD5 (*rth*6)-Fw	5´ CTGGGTGCTTGCTCATCTGT 3´
ZmCslD5 (*rth*6)-Rev	5´ GTTGGCAGCTTGTGATGGAG 3´
Myosin-Fw	5´ AGAAGGCCGTACAGGATCTTACC 3´
Myosin-Rev	5´ CAAGGAGAGACTCTGTGAGCTTCA 3´
